# Mapping the chromatin landscape and Blimp1 transcriptional targets that regulate trophoblast differentiation

**DOI:** 10.1038/s41598-017-06859-9

**Published:** 2017-07-28

**Authors:** Andrew C. Nelson, Arne W. Mould, Elizabeth K. Bikoff, Elizabeth J. Robertson

**Affiliations:** 1 Sir William Dunn School of Pathology, University of Oxford, South Parks Road, Oxford, OX1 3RE UK; 2 School of Life Sciences, Gibbet Hill Campus, University of Warwick, Coventry, CV4 7AL UK

## Abstract

Trophoblast stem cells (TSCs) give rise to specialized cell types within the placenta. However, the regulatory mechanisms that guide trophoblast cell fate decisions during placenta development remain ill defined. Here we exploited ATAC-seq and transcriptional profiling strategies to describe dynamic changes in gene expression and chromatin accessibility during TSC differentiation. We detect significantly increased chromatin accessibility at key genes upregulated as TSCs exit from the stem cell state. However, downregulated gene expression is not simply due to the loss of chromatin accessibility in proximal regions. Additionally, transcriptional targets recognized by the zinc finger transcriptional repressor *Prdm1*/Blimp1, an essential regulator of placenta development, were identified in ChIP-seq experiments. Comparisons with previously reported ChIP-seq datasets for primordial germ cell-like cells and E18.5 small intestine, combined with functional annotation analysis revealed that Blimp1 has broadly shared as well as cell type-specific functional activities unique to the trophoblast lineage. Importantly, Blimp1 not only silences TSC gene expression but also prevents aberrant activation of divergent developmental programmes. Overall the present study provides new insights into the chromatin landscape and Blimp1-dependent regulatory networks governing trophoblast gene expression.

## Introduction

The placenta, a specialized organ comprised of both maternal and foetal tissues is essential to support mammalian embryonic development^1^. Most primates and rodents form a hemochorial placenta, where maternal blood directly contacts foetal trophoblasts derived from the outer layer of the blastocyst, the so-called trophectoderm. Following implantation the trophectoderm gives rise to both the extraembryonic ectoderm (ExE) lying proximal to the epiblast, as well as the ectoplacental cone (EPC) which forms on the distal maternal interface. The ExE contains the progenitors of specialized syncytiotrophoblasts that mediate maternal-foetal exchange in the placental labyrinth region, whereas the EPC gives rise to the outer spongiotrophoblast (SpT) layer - the source of diverse trophoblast giant cells (TGCs) including the spiral artery-associated trophoblast giant cells (SpA-TGCs) that invade the maternal uterine tissue and replace arterial linings to promote increased blood flow to the foetus^2, 3^.

Trophoblast stem cells (TSCs) isolated from the trophectoderm or the early post-implantation (E6.5) ExE retain self-renewal capacity in the presence of FGF4 and TGFβ1^4, 5^. TSCs are lineage-restricted and when reintroduced into blastocysts exclusively populate the labyrinth and SpT compartments of the placenta^6^. Upon growth factor withdrawal, TSCs differentiate into mature trophoblast subtypes. In contrast, pluripotent embryonic stem cells (ESCs) derived from the blastocyst inner cell mass, differentiate to form tissues of the embryo proper. The regulatory mechanisms that govern gene expression changes during ESC differentiation are well characterized^7^. Pluripotent ESCs have relatively low levels of heterochromatin, and lineage restriction during differentiation is accompanied by chromatin condensation^7^. Genome-wide histone ChIP and DNase I hypersensitivity approaches have been used to describe the global chromatin landscape in TSCs^8–10^. However, the dynamic epigenetic changes that co-ordinately regulate local and long-distance promoter-enhancer interactions governing trophoblast differentiation remain ill defined.

Originally discovered as a master regulator of plasma cell differentiation^11^, Blimp1 (encoded by the *Prdm1* gene) governs cell-fate decisions in the embryo and adult organism. Blimp1 is required for primordial germ cell specification^12, 13^ and plays essential roles in the epidermis^14, 15^, mammary gland development^16^, and post-natal reprogramming of intestinal enterocytes^17, 18^. *Prdm1*/Blimp1 null embryos die at around E10.5 due to placental insufficiency^13^. *Prdm1* is broadly expressed throughout the EPC and SpT layer^19^. The lethality is associated with loss of the Blimp1-dependent invasive SpA-TGC lineage^19^. During *in vitro* differentiation Blimp1 expression is detectable in day 2 diploid trophoblasts, and later in mature TGCs^20^. The *Prdm1* loss-of-function phenotype is recapitulated *in vitro*, since *Prdm1* null TSCs display restricted differentiation abilities and fail to upregulate SpA-TGC markers^20^.

To learn more about transcriptional and epigenetic mechanisms controlling maturation of various trophoblast cell types, here we exploited ATAC-seq technology^21^ to describe global chromatin accessibility changes associated with loss of the stem cell state and the emergence of the Blimp1+ trophoblast cell lineage. Additionally, our ChIP-seq and transcriptional profiling experiments identified numerous candidate target genes directly repressed by Blimp1 during trophoblast differentiation. Combined with functional annotation analysis and comparisons with published microarray datasets, collectively the present genome-wide analyses reveal key features of the chromatin landscape controlling trophoblast gene expression profiles and advance our understanding of the signalling pathways that regulate development of the trophoblast cell lineage.

## Results

### A subset of TSC cis-regulatory elements are detectable at the 8-cell stage

**Figure 1:**
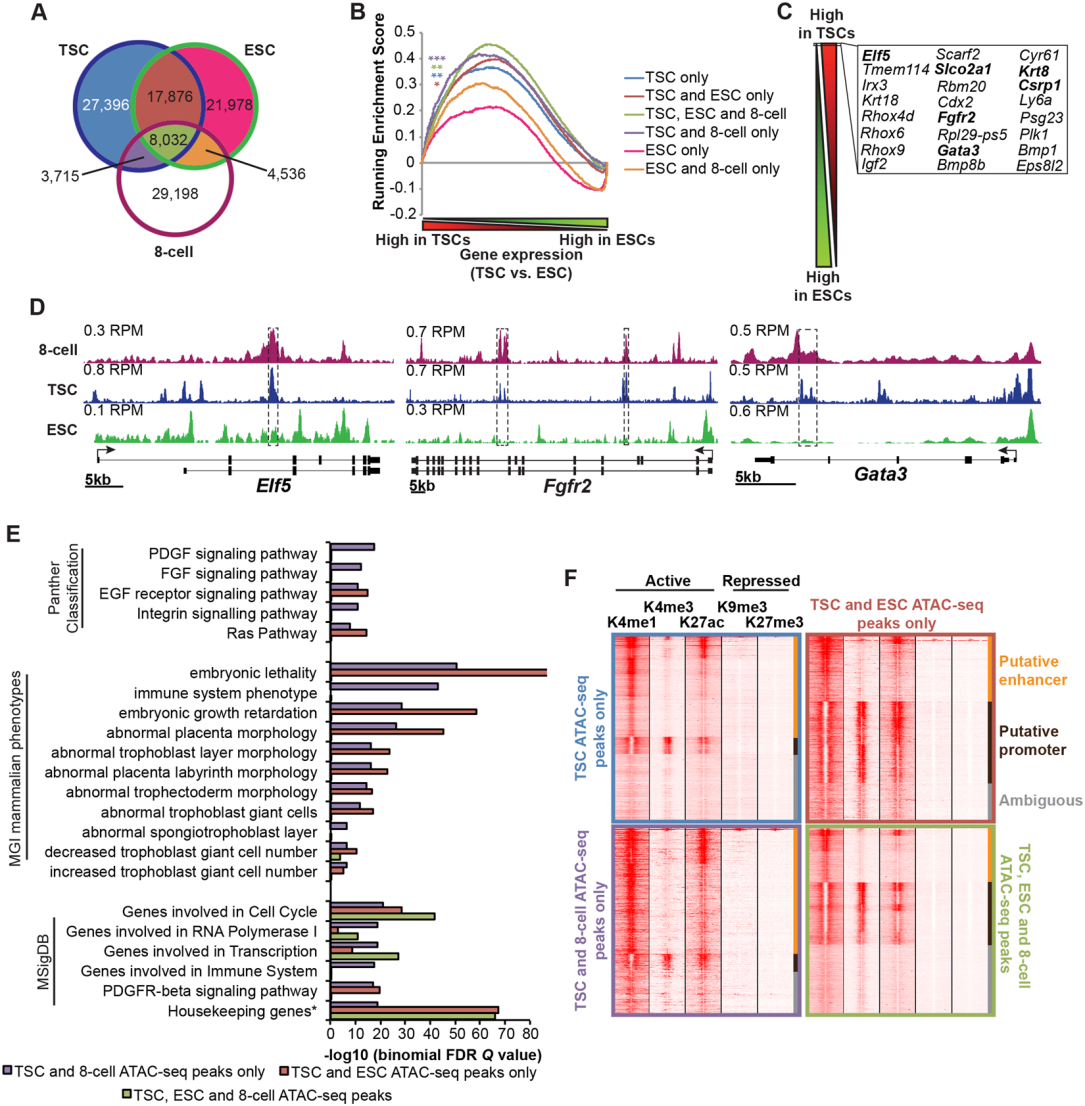
A subset of TSC *cis*-regulatory elements is apparent at the 8-cell stage. (**A**) Partially overlapping TSC, ESC and 8-cell ATAC-seq peaks. See also Supplementary Figure S2. (**B**) Gene Set Enrichment Analysis comparing TSC and ESC gene expression microarrays with each subset of ATAC-seq peaks shown above in A (nearest TSS ±100 kb from each peak annotated). *P ≤ 2 × 10^−2^, FWER *P* ≤ 1 × 10^−2^; **P ≤ 1 × 10^−2^, FWER *P* ≤ 1 × 10^−2^; ***P ≤ 1 × 10^−3^, FWER *P* ≤ 3 × 10^−3^. (**C**) Top 24 genes with significantly higher expression (by fold change and adjusted *P* value < 1 × 10^−9^ – see Supplementary Table S2) in TSCs vs. ESCs. Genes with regions selectively accessible in both TSCs and 8-cell embryos are in bold. (**D**) ATAC-seq tracks at *Elf5*, *Fgfr2* and *Gata3*. Regions selectively accessible in both TSCs and 8-cell embryos are boxed. RPM = Reads per million. (**E**) TSC ATAC-seq peaks shared with 8-cell embryos occur proximal to genes with functions consistent with placenta development and are relatively underenriched for housekeeping functions. Analysis of TSC ATAC-seq subsets indicated in Fig. 1A using GREAT^84^. The nearest TSS ±100 kb from each peak was annotated. Selected Panther, MGI phenotypes and MSigDB terms are shown. * “Housekeeping genes identified as expressed across 19 normal tissues”. Note: ATAC-seq peak subsets not represented e.g. unique TSC peaks are too large for statistically meaningful analysis by GREAT^84^. (**F**) Heatmap of H3K4me1, H3K4me3, H3K27ac, H3K9me3 and H3K27me3 ChIP-seq data at subsets of TSC ATAC-seq peaks as defined by Fig. 1A. Broadly, H3K4me1, H3K4me3, H3K27ac are considered to be “active” histone marks, with H3K4me1 marking enhancer and H3K4me3 promoters, while H3K9me3 and H3K27me3 are repressive marks^88^. TSC ATAC-seq peaks are defined at “putative enhancer” based on strong H3K4me1 signal relative to H3K4me3, and “putative promoter” based on strong H3K4me3 signal.

ESCs and TSCs display distinct expression profiles and developmental capabilities^5, 22^. To map *cis*-regulatory elements (CREs) in TSCs we performed ATAC-seq experiments, identifying 57,019 distinct regions of accessible chromatin (Supplementary Fig. S1). Then to test whether these might be cell-type specific or possibly pre-established at an earlier embryonic stage we compared our TSC ATAC-seq with 8-cell stage embryo and ESC datasets^23^. We found that ~45% of TSC ATAC-seq peaks were shared with ESCs, as opposed to ~20% shared with the 8-cell dataset (Fig. 1A, Supplementary Table S1, Supplementary Fig. S2). Additionally, comparisons with expression microarrays (Supplementary Table S2) revealed that TSC ATAC-seq peaks, including those shared with 8-cell embryos are consistently associated with high levels of TSC expression (Fig. 1B). Moreover, TSC and 8-cell ATAC-seq peaks were present in regions located near known TSC marker genes, such as *Elf5*, *Fgfr2* and *Gata3* (Fig. 1C,D). Functional annotation analysis of genes with ATAC-seq peaks strongly expressed in TSCs demonstrates enrichment for placental genes and signalling pathways, including the TGF-beta pathway known to promote trophoblast self renewal^4^, as well as EGFR1 and IL-1 signalling, which mediate trophoblast invasion^24, 25^ (Supplementary Fig. S3). Importantly, analysis of the subsets of peaks detected in both TSCs and 8-cell embryos but not ESCs indicates significant enrichments for genes with functions relevant to placenta development, suggesting they do not simply represent a subset housekeeping functions (Fig. 1E). To determine whether TSC ATAC-seq peaks overlapping with 8-cell and ESC data may represent distinct classes of CRE we used published TSC histone modification data^9^. This revealed that a high proportion of TSC ATAC-seq peaks, including those shared with 8-cell embryos are putative enhancers (H3K4me1/H3K27ac positive), while those shared with ESCs are more likely to be promoters (H3K4me3/H3K27ac positive; Fig. 1F). Consistent with this, overlapping TSC/8-cell ATAC-seq peaks are typically further from TSSs than overlapping TSC/ESC peaks (Supplementary Fig. S4). Collectively these results identify putative CREs controlling TSC fate, and demonstrate a subset of TSC enhancers are detectable at the 8-cell stage.

### Upregulated gene expression during trophoblast differentiation reflects selective expansion of accessible chromatin regions

**Figure 2:**
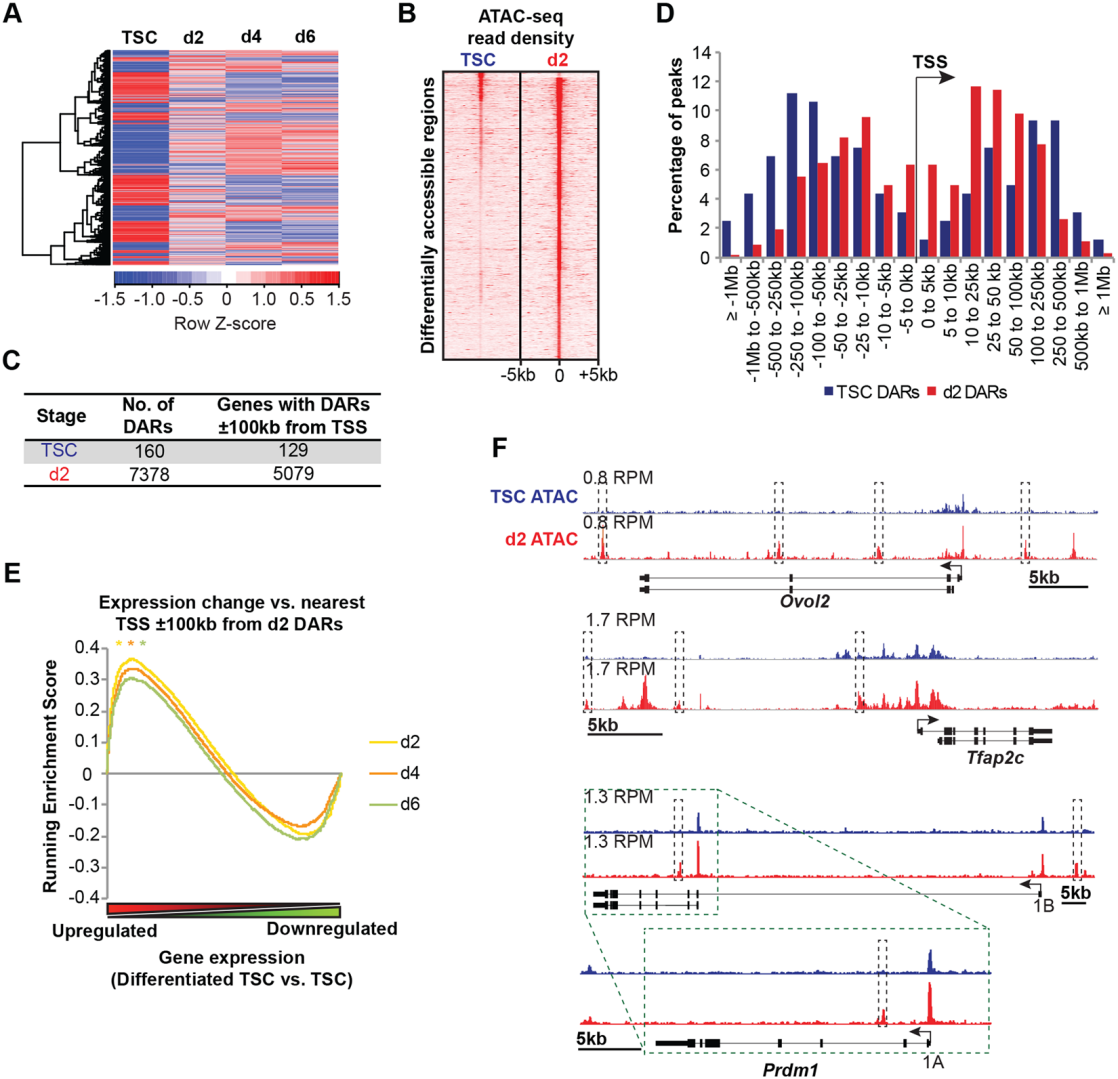
Increased chromatin accessibility leads to activated gene expression during trophoblast differentiation. (**A**) Microarray heatmap of genes differentially expressed during trophoblast differentiation. In contrast to our previous study where we used these data to identify genes differentially expressed between wild type and *Prdm1* mutant trophoblasts^20^, here we present gene expression changes during wild type TSC differentiation. (**B**) Heatmap of ATAC-seq read densities at high-confidence TSC and d2 DARs as indicated in B. (**C**) Defined differentially accessible regions (DARs) and corresponding nearest TSSs ±100 kb. (**D**) Distribution of DARs relative to the nearest TSS defined by GREAT^84^. TSC DARs are significantly further from TSSs than d2 DARs – *P* = 6.0 × 10^−8^, two-tailed homoscedastic t-test. (**E**) Gene Set Enrichment Analysis comparing genes with d2 DARs (nearest TSS ±100 kb) and microarray gene expression changes during TSC differentiation. *P ≤ 1 × 10^−3^. (**F**) TSC and d2 ATAC-seq peaks at the *Ovol2*, *Tfap2c* and *Prdm1* loci. RPM = Reads per million. Identified d2 DARs are indicated in grey boxes. Distinct annotated *Prdm1* promoters are indicated.

**Figure 3:**
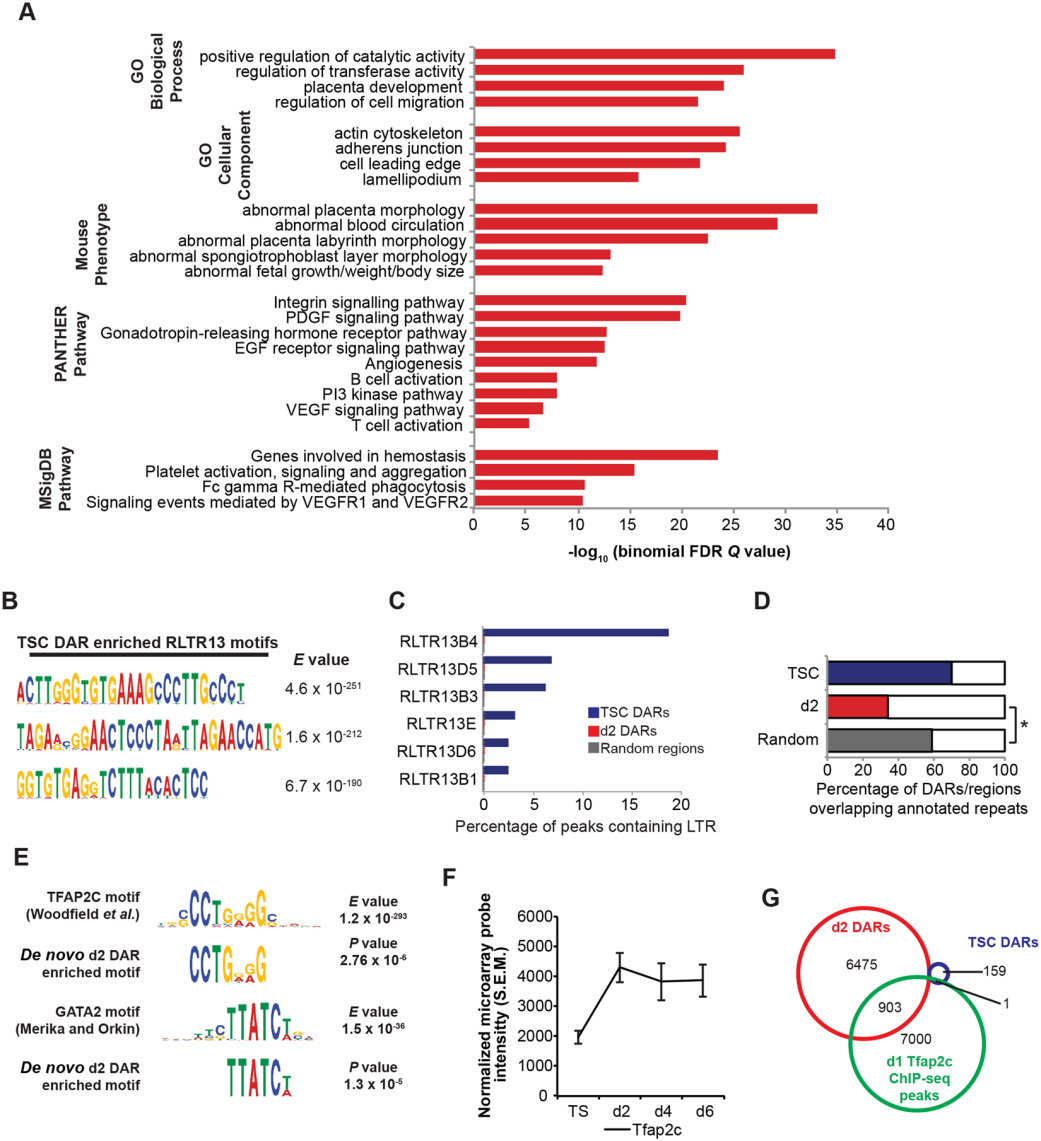
Regulation of trophoblast function and placenta development by genomic loci showing changes in chromatin accessibility during TSC differentiation. (**A**) Functional annotation analysis of genes with d2 DARs (nearest TSS ±100 kb) using GREAT^84^. (**B**) Sequence motifs identified in TSC DARs using MEME-ChIP and associated *E* value confidence scores. (**C**) Comparison of RLTR13 family repeats located within TSC and d2 DARs, and random genomic regions. (**D**) The percentages of TSC and d2 DARs, and random genomic regions overlapping annotated repeats. Significant under-representation of repeat regions in d2 DARs was determined using Chi-square test. *P = 7.3 × 10^−217^. (**E**) Sequence motifs identified in d2 DARs using MEME-ChIP aligned with published Tfap2c and GATA2 consensus binding motifs^89, 90^. Both *E* value confidence scores for *de novo* motif identification and *P* value for match to published motif are shown. (**F**) Microarray expression profile of Tfap2c during wild type trophoblast differentiation. (**G**) Overlap of TSC and d2 DARs with published Tfap2c ChIP-seq peaks^31^. See also Supplementary Figure S2.

Dramatic changes in gene expression profiles were detectable during TSC differentiation (Fig. 2A). To investigate chromatin accessibility we performed ATAC-seq experiments at day 2 of differentiation (d2). When TSC and d2 ATAC-seq datasets were compared we identified thousands of high-confidence differentially accessible regions (DARs, see Methods; Fig. 2B,C, Supplementary Tables S3 and 4, Supplementary Fig. S1). We refer to regions with enhanced accessibility at d2 as d2 DARs, and those with enhanced accessibility in TSCs as TSC DARs. The majority of d2 DARs are located 5–100 kb away from the nearest TSS. In contrast, TSC DARs are located in more distal regions (Fig. 2D, Supplementary Fig. S5). Gene Set Enrichment Analysis (GSEA) of d2 DARs revealed significant enrichment at genes with upregulated expression in d2, d4 or d6 TGCs (Fig. 2E). For example, as shown in Figs 2F and 3A, d2 DARs are located proximal to a previously described regulator of trophoblast cell migration *Ovol2* ^26^, *Tfap2c* known to govern trophoblast differentiation and proliferation^27–29^, and *Prdm1* required for SpA-TGC specification^19^. These observations strongly suggest that increased accessibility promotes activation of proximal gene expression. Functional annotation analysis revealed a highly significant enrichment for placental phenotypes and pathways, including cell migration, PDGF signalling pathway that maintains the placental haematopoietic niche^30^, as well as genes controlling spongiotrophoblast and labyrinth development and morphology (Fig. 3A).

To further characterize these putative CREs we performed *de novo* motif analysis. Interestingly, TSC DARs were highly enriched for multiple long sequence motifs corresponding to regions of the RLTR13 family endogenous retroviruses (ERVs) (Fig. 3B,C, Supplementary Fig. S5). In contrast, d2 DARs are significantly depleted for annotated repeat regions (Fig. 3D) but were found to be enriched for consensus binding motifs recognized by several key transcriptional regulators implicated in trophoblast lineage development, including Tfap2c, and Ets and Gata factors (Fig. 3E, Supplementary Fig. S6). Though TSCs express Tfap2c, our microarray data suggests that it is upregulated during early differentiation (Fig. 3F). Interestingly, comparison with a published Tfap2c ChIP-seq dataset from d1 differentiated TSCs^31^ revealed a subset of d2 DARs display Tfap2c occupancy (Fig. 3G, Supplementary Table S4), strongly suggesting that Tfap2c-mediated induction of trophoblast differentiation^31^ occurs in part through activation of newly accessible CREs.

### Silencing of TSC gene expression reflects downregulation of the Fgf-regulated transcription factor Esrrb

**Figure 4:**
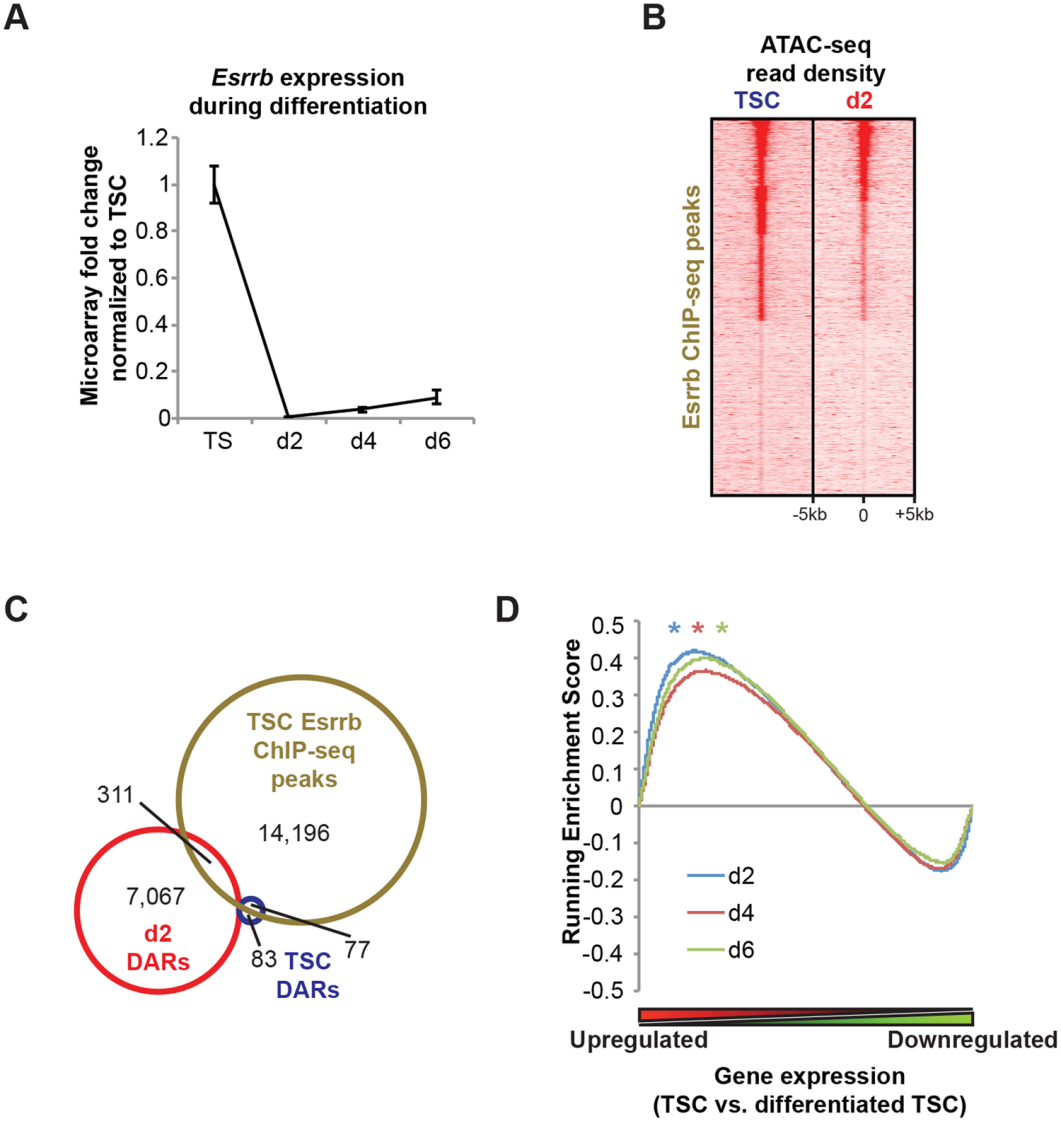
TSC gene expression controlled by the Fgf-dependent transcription factor Esrrb. (**A**) Microarray analysis of *Esrrb* expression during TSC differentiation. Fold change relative to TSC is shown. Data represents means ±S.E.M., n ≥ 8 per stage. (**B**) Heatmap of TSC and d2 ATAC-seq data at Esrrb TSC ChIP-seq coordinates. (**C**) Overlap of Esrrb ChIP-seq peaks with TSC and d2 DARs. See also Supplementary Figure S2. (**D**) Gene Set Enrichment Analysis comparing Esrrb ChIP-seq peaks and microarray changes in gene expression at three stages of *in vitro* differentiation compared with TSCs. *P < 1 × 10^−3^.

Results above demonstrate that activation of gene expression during TSC differentiation is accompanied by increased chromatin accessibility. However, downregulated gene expression does not appear to be consistently associated with reduced chromatin accessibility (Supplementary Fig. S1). Loss of the pivotal regulator Esrrb accompanies TSC differentiation induced by FGF4 withdrawal^5^ (Fig. 4A). To further explore a possible relationship to the downregulation of TSC gene expression we analyzed a published Esrrb ChIP-seq dataset^32^. Esrrb occupancy in TSCs was detectable at chromatin accessible in both TSCs and d2 cell populations (Fig. 4B,C, Supplementary Tables S3 and 4). However, Esrrb binding in TSCs was markedly enriched at genes characterized by downregulated expression during differentiation (Fig. 4D). Thus downregulated TSC gene expression in part reflects loss of Esrrb occupancy.

### Identification of Blimp1 target genes

**Figure 5:**
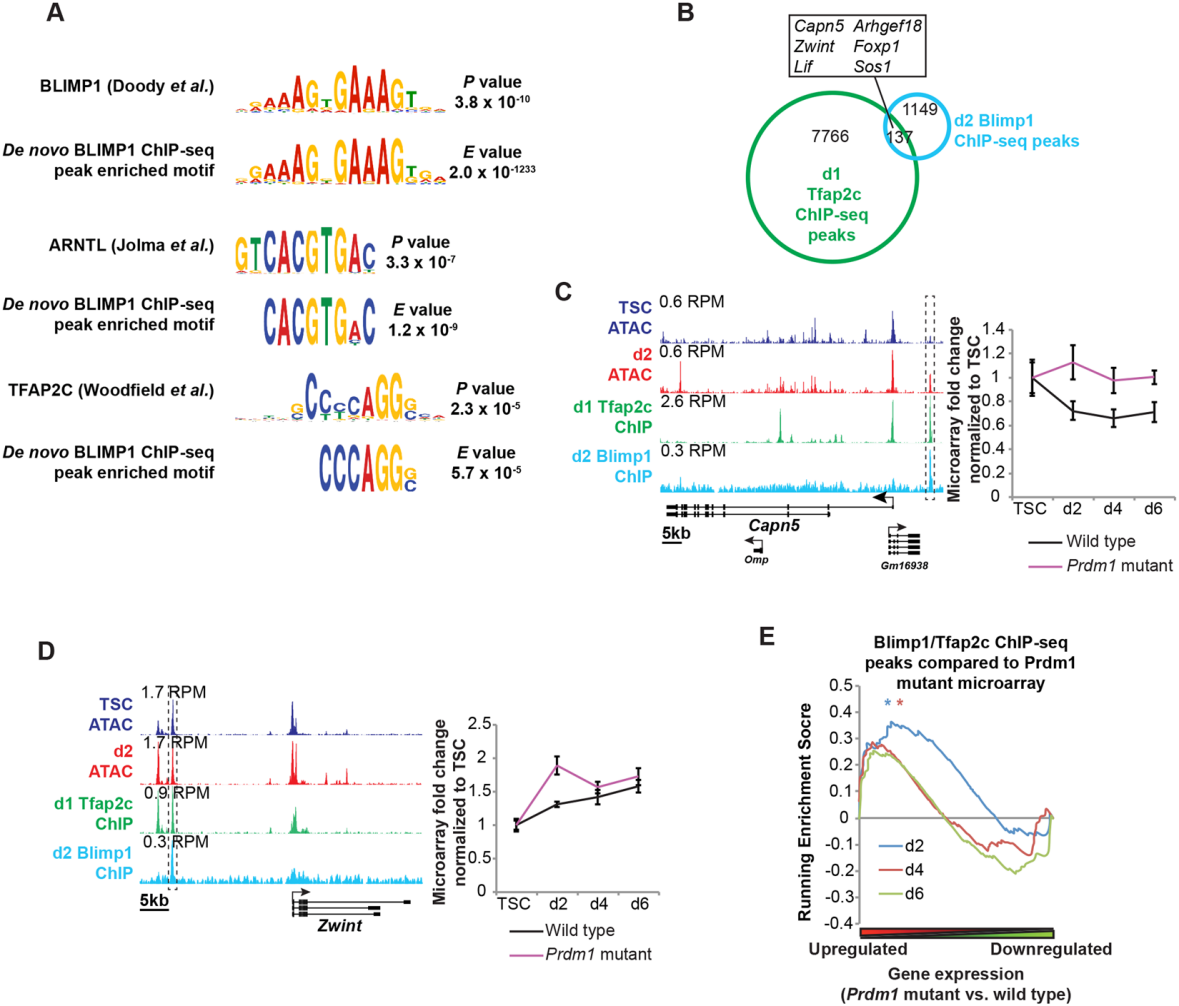
A subset of Blimp1-bound regions may also display Tfap2c occupancy. (**A**) MEME-ChIP analysis of d2 Blimp1 ChIP-seq peaks identified multiple transcription factor binding motifs^89, 91, 92^. *E* value confidence scores for *de novo* motif identification and *P* value for match to published motif are shown. (**B**) Overlap of d2 Blimp1 and d1 Tfap2c ChIP-seq peaks. Examples of genes occupied by both Blimp1 and Tfap2c at overlapping coordinates ±100 kb of their TSS are indicated. See also Supplementary Figure S2. (**C**,**D**) Comparison of ATAC-seq and ChIP-seq peaks showing Blimp1 and Tfap2c occupancy at *Capn5* (**C)** and *Zwint* (**D**), with microarray expression profiles. Fold change relative to TSC is shown. Data represents means ±S.E.M., n ≥ 6 per genotype per stage. RPM = Reads per million. Gene Set Enrichment Analysis comparing overlapping Blimp1/Tfap2c ChIP-seq peaks and microarray changes in gene expression during TSC differentiation. *P < 5 × 10^−2^.

We previously described essential contributions made by the transcriptional repressor Blimp1 during placental development^19^. Recent single-cell RNA-seq expression profiling experiments have identified functionally distinct Blimp1+ trophoblast cell types^20^. To characterize Blimp1 transcriptional targets in differentiating TSCs, here we performed ChIP-seq at d2, when Blimp1 is strongly expressed in diploid progenitor trophoblasts^20^. We identified 1286 ChIP-seq peaks that were strongly enriched for the Blimp1 binding consensus motif. Many of these target sites also contain additional transcription factor binding motifs including those corresponding to Tfap2c and Arntl (Fig. 5A).

Blimp1 and Tfap2c function collaboratively as key components of a tripartite transcription factor network that regulates primordial germ cell (PGC) specification through CRE co-occupancy^33^. To examine this interaction in trophoblasts we compared our Blimp1 target sites with the trophoblast Tfap2c ChIP-seq dataset^31^. Interestingly we found a subset of putative CREs that could possibly be bound by either or both Blimp1 and Tfap2c, strongly suggesting that this transcriptional partnership also regulates gene expression in trophoblasts (Fig. 5B–D, Supplementary Table S5). Moreover, microarray analysis indicates that many of these Blimp1 and Tfap2c occupied sites are associated with upregulated gene expression in *Prdm1* mutants (which lack the Blimp1 protein) (Fig. 5C–E).

**Figure 6:**
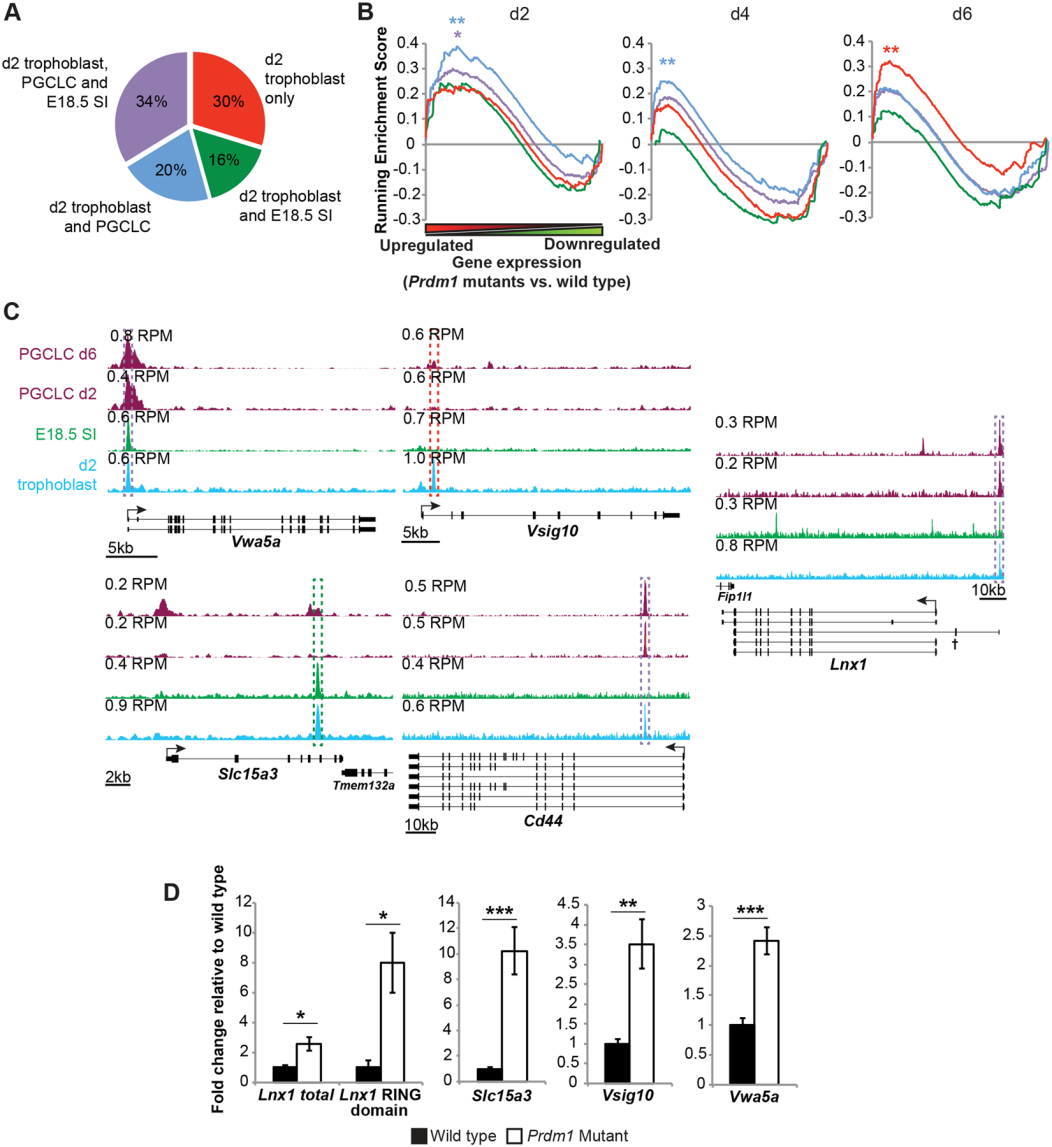
ChIP-seq identifies common and cell type-specific Blimp1 target genes. (**A**) Partially shared Blimp1 ChIP-seq peaks in d2 trophoblast, E18.5 small intestine (SI)^18^ and primordial germ cell like cells (PGCLCs)^34^. (**B**) Gene Set Enrichment Analysis of wild type and *Prdm1* mutant trophoblast gene expression profiles at day 2, 4 and 6 of differentiation in comparison with Blimp1 ChIP-seq peaks (nearest TSS ±100 kb) categorized as in A. *P ≤ 5 × 10^−3^, FWER *P* ≤ 5 × 10^−2^; **P ≤ 1 × 10^−3^, FWER *P* ≤ 1 × 10^−3^. (**C**) ChIP-seq tracks showing peaks shared between different cell types (*Vwa5a*, *Slc15a3, Cd44*, *Lnx1*) or unique to d2 trophoblasts (*Vsig10*). Peaks are boxed and colour-coded as in panel A. † = *Lnx1* exon encoding the RING domain. (**D**) RT-qPCR validation of expression changes in wild type and *Prdm1* mutant d2 trophoblasts. *P ≤ 5 × 10^−3^; **P ≤ 1 × 10^−3^; ***P ≤ 3 × 10^−5^. Data represents means ±S.E.M., n ≥ 9 per genotype.

Next we compared our d2 ChIP-seq peaks to published datasets from PGC-like cells (PGCLCs)^34^ and E18.5 small intestine (SI)^18^. The majority of our ChIP-seq peaks are also detectable in either the SI or PGCLC datasets. However, roughly 30% were exclusively present in the d2 trophoblasts (Fig. 6A, Supplementary Table S5). Functional annotation analysis similarly suggests that Blimp1 has both unique and common roles shared amongst these cell types (Supplementary Fig. S7).

Comparisons of transcriptional profiles from *Prdm1* mutant vs. wild type TGCs at d2, d4 and d6 of differentiation^20^ with our ChIP-seq data identified 125 candidate target genes represented amongst the upregulated transcripts (Fig. 6B, Supplementary Table S6). The list includes the specific isoform of *Lnx1* exhibiting E3 ubiquitin ligase activity^35^, candidate tumour suppressor *Vwa5a* ^36^, a histidine transporter *Slc15a3*, novel immunoglobulin domain containing gene *Vsig10*, and *Cd44* – a cell surface receptor associated with trophoblast invasion^37^. Representative ChIP-seq peaks and qPCR validation of expression changes are shown in Fig. 6C,D.

**Figure 7:**
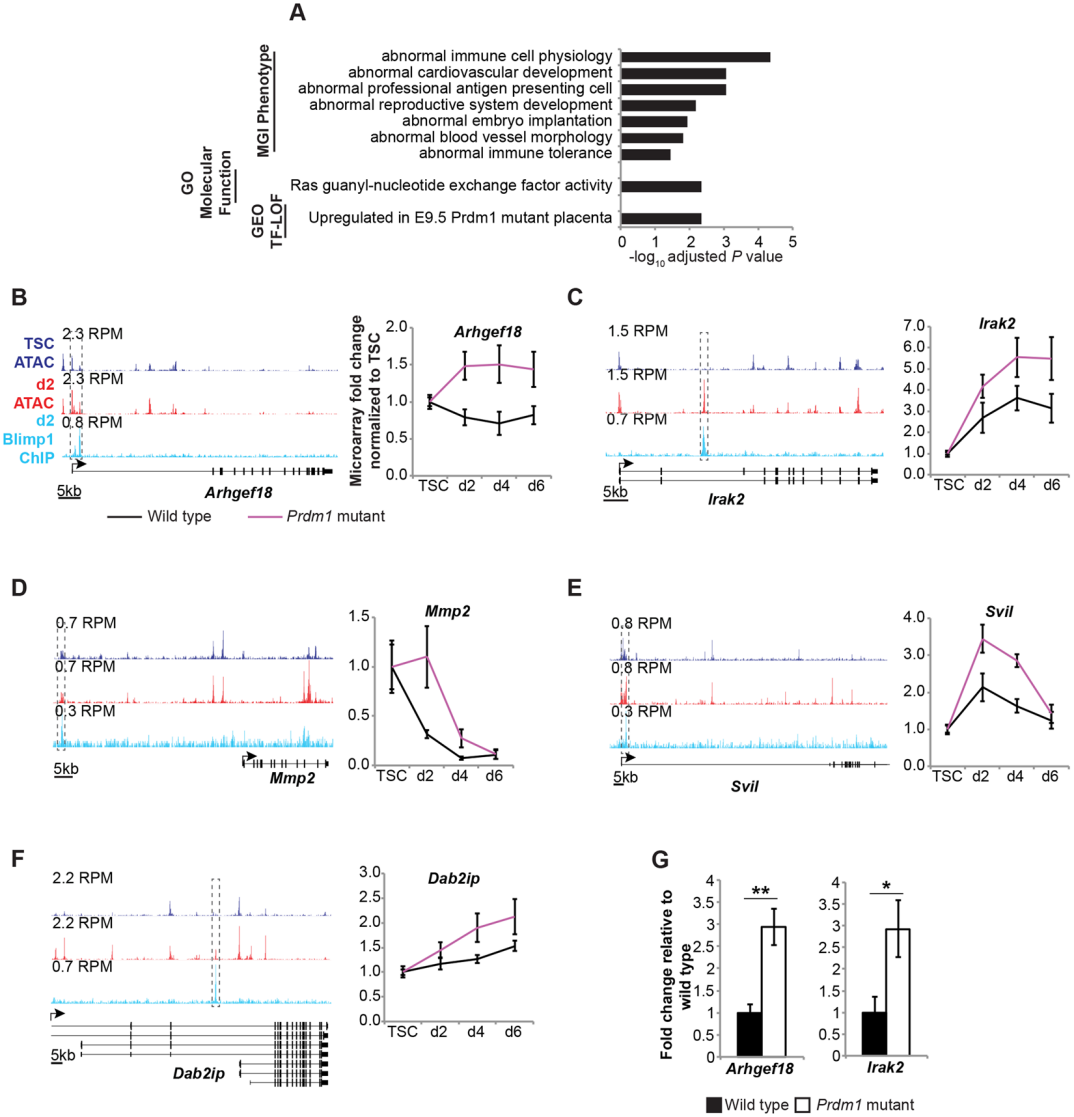
Blimp1 target genes govern diverse processes including immunity, vascularity, signalling and invasion. (**A**) Functional annotation analysis of genes with Blimp1 ChIP-seq peaks (nearest TSS ±100 kb) that are upregulated in *Prdm1* mutant trophoblasts (*P* ≤ 0.05, fold change ≥ 1.5) using Enrichr^85^ classified according to Mouse Genome Informatics Phenotype, Gene Ontology Molecular Function and NCBI transcription factor loss-of-function terminology. (**B**–**F**) Comparison of ATAC-seq and Blimp1 ChIP-seq tracks with microarray expression profiles. Data represents means ±S.E.M., n ≥ 6 per genotype per stage. RPM = Reads per million. (**G**) qRT-PCR validation of Blimp1 target gene expression. *P = 2 × 10^−2^; **P = 5 × 10^−4^.

Functional annotation analysis of the 125 genes showing both Blimp1 binding ±100 kb of TSS and upregulated expression in *Prdm1* mutant TGCs (≥1.5-fold, *P* ≤ 0.05; Supplementary Table S6) demonstrates associations with defective vascular remodelling and abnormal immunological functions (Fig. 7A). Strikingly, we also discovered significant overlaps between the Blimp1 targets identified here in *in vitro* differentiated trophoblasts and genes upregulated in E9.5 *Prdm1* mutant placenta^19^ (Fig. 7A), such as *Svil* and *Dab2ip* known to regulate cell migration and invasion (Fig. 7E,F). Representative results are shown in Fig. 7B–F and qPCR validation in Fig. 7G.

### Blimp1 represses TSC gene expression and prevents aberrant differentiation

**Figure 8:**
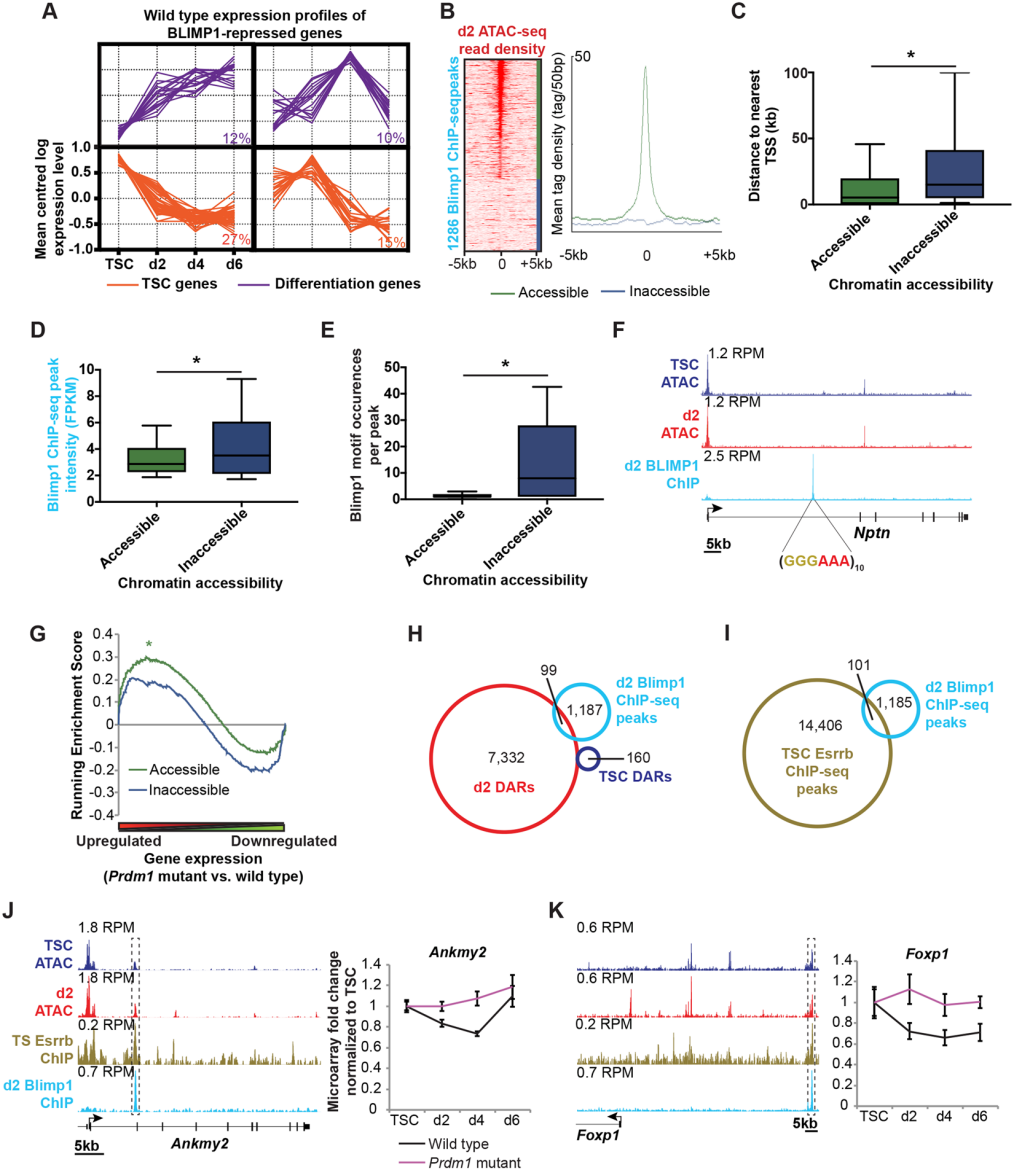
Blimp1 represses TSC gene expression and prevents aberrant induction of differentiation genes. (**A**) K-means clustering of microarray expression profiles of the 125 identified Blimp1 target genes during TSC differentiation. Blimp1 direct target genes include both TSC-expressed genes (orange) and those activated during differentiation (purple). Percentages of genes within each cluster are indicated. (**B**) Heatmap and density plot of d2 ATAC-seq reads at Blimp1 ChIP-seq peaks distinguish Blimp1 occupancy at accessible or inaccessible target sites. (**C**) Distance of Blimp1 ChIP-seq peaks from the nearest TSS at accessible or inaccessible chromatin regions categorized as in panel B. Blotplot intervals indicate median, 10^th^, 25^th^, 75^th^ and 90^th^ percentiles. Statistical significance was calculated using two-tailed heteroscedastic t-test. *P = 1.1 × 10^−14^. (**D**) Blimp1 ChIP-seq peak intensities at accessible or inaccessible chromatin regions. Statistical significance was calculated using two-tailed heteroscedastic t-test. FPKM = fragments per kilobase per million reads; *P = 2.4 × 10^−6^. (**E**) Occurrence of the Blimp1 consensus binding motif located within Blimp1 ChIP-seq peaks at accessible or inaccessible chromatin regions. Statistical significance was calculated using two-tailed heteroscedastic t-test. *P = 1.0 × 10^−44^. (**F**) Blimp1 occupancy at closed chromatin corresponds to low complexity repeats homologous to the Blimp1 consensus binding motif. RPM = Reads per million. (**G**) Gene Set Enrichment Analysis of wild type and *Prdm1* mutant trophoblast gene expression profiles at day 2 of differentiation in comparison with Blimp1 ChIP-seq peaks at accessible and inaccessible chromatin (nearest TSS ±100 kb annotated). *P < 1 × 10^−3^, FWER *P* = 5 × 10^−3^. (**H**) Overlap of d2 Blimp1 ChIP-seq peaks with TSC and d2 DARs. See also Supplementary Figure S2. (**I**) Overlap of d2 Blimp1 ChIP-seq peaks with TSC Esrrb ChIP-seq peaks. See also Supplementary Figure S2. (**J**,**K**) Blimp1 and Esrrb occupancy at shared ATAC-seq and ChIP-seq peaks in comparison with microarray expression profiles for *Ankmy2* (**J**) and *Foxp1* (**K**). Data represents means ±S.E.M., n ≥ 6 per genotype per stage. RPM = Reads per million.

During B cell maturation Blimp1 silences ongoing expression of genes such as *Pax5*, *Ciita* and *Myc*, to promote terminal plasma cell differentiation^11, 38^. However, Blimp1 occupancy in the developing gut prevents premature activation of genes associated with the adult metabolic signature^17^. Expression profiling of the 125 Blimp1 targets during TSC differentiation demonstrates genes highly expressed in TSCs and also genes induced during differentiation (Fig. 8A). Thus *Prdm1*/Blimp1, appears to become activated to repress a subset of TSC genes, such as *Capn5*, *Mmp2*, *Ankmy2* and *Foxp1* (Figs 5C,7D and 8J,K), while also inhibiting expression of genes induced during differentiation such as *Zwint*, *Irak2* and *Dab2ip* (Figs 5D and 7C,F). Thus during SpA-TGC specification Blimp1 probably has dual functions to silence both TSC and aberrant lineage-specific gene expression.

Recent experiments suggest that Blimp1 binds both open and closed chromatin in plasmablasts^39^. Comparison of our ATAC-seq data with Blimp1 ChIP-seq peaks revealed graded degrees of chromatin accessibility at occupied sites, and approximately a third that appear to be inaccessible (Fig. 8B). The Blimp1 ChIP-seq peaks at accessible regions tend to be closer to TSSs (Fig. 8C). Blimp1 ChIP-seq peak intensities are, however, slightly greater at inaccessible chromatin (Fig. 8D). Notably, this subset of ChIP-seq peaks tend to contain low complexity repeat sequences including multiple copies of the Blimp1 binding consensus (Fig. 8E,F), likely to enhance the probability of binding. Importantly, however, while Blimp1 binding at accessible chromatin is significantly correlated with genes upregulated in mutant trophoblasts, binding at inaccessible chromatin is not (Fig. 8G). It is therefore tempting to speculate that binding within these distal repeat-enriched regions does not represent a key functional requirement.

Only 99 of the 1286 Blimp1-occupied regions in d2 trophoblasts showed strongly enhanced accessibility on differentiation (i.e. binding at d2 DARs) (Fig. 8H). Similarly, only 15 of the 125 candidate target genes, notably *Irak2* and *Dab2ip* display occupancy within d2 DARs (Fig. 7C,F). Other Blimp1 target sites at constitutively accessible chromatin include a subset occupied by Esrrb in TSCs, such as at *Ankmy2* and *Foxp1* (Fig. 8I–K). It therefore seems likely that expression of a subset of genes maintained by Esrrb in TSCs is silenced by Blimp1 during differentiation via the same CREs.

In sum results above characterize structural changes at discrete chromatin regions associated with trophoblast differentiation, and identify novel targets genes that potentially regulate Blimp1-dependent trophoblast lineage specification.

## Discussion

The epigenetic landscape and transcriptional networks that govern self-renewal and maintenance of the stem cell state have been extensively studied in TSCs and ESCs^7, 9, 31, 32, 40^. However, relatively little is known about epigenetic mechanisms governing TSC differentiation and specification of mature trophoblast subtypes.

The present ATAC-seq experiments examine genome-wide changes in chromatin accessibility during trophoblast differentiation. We identified thousands of DARs present at early stages of TSC differentiation, as well as regions that distinguish TSCs from ESCs. Remarkably, a subset of these is detectable as early as the 8-cell stage embryo. Recent experiments demonstrate that apical-basal cell polarity leading to segregation of the trophectoderm and inner cell mass becomes apparent at the 8-cell stage^41^. It is tempting to speculate that formation of the apical domain during the establishment of the trophectoderm fate may occur via accessible chromatin domains common to 8-cell embryos and TSCs that have been identified here.

In marked contrast to ESCs, TSC exit from the stem cell-state is associated with globally increased rather than decreased chromatin accessibility. Expansion of accessible chromatin is associated with activated gene expression during differentiation. Downregulated gene expression does not simply reflect formation of highly inaccessible chromatin by day 2 of differentiation. Rather trophoblast differentiation initiated by the withdrawal of FGF4 and TGFβ1 leads to loss of key transcription factors (including Cdx2, Eomes and the growth factor-dependent Esrrb). Moreover, we found that Esrrb ChIP-seq peaks in TSCs are significantly associated with genes downregulated during TSC differentiation. Thus gene expression changes during TSC differentiation can be explained in large part due to the concomitant loss of Esrrb^32^. Interestingly, a subset of Blimp1 targets are present at CREs previously occupied by Esrrb, including *Ankmy2*, *Foxp1* and others (Fig. 8J,K). Blimp1 also represses the expression of a subset of genes induced during differentiation, through both constitutively accessible regions (e.g. *Zwint* Fig. 5D) and those that only become accessible during differentiation (e.g. *Irak2* and *Dab2ip* Fig. 7C,F). Thus Blimp1 appears to have dual repressive roles – silencing pre-existing gene expression as well as preventing aberrant induction of gene expression.

Both the structural and functional characteristics of the placenta display considerable diversity across mammalian species^42^. Considerable evidence suggests that species-specific ERVs provide crucial regulatory signals driving rapid evolution through re-wiring transcriptional networks in TSCs^9^. Here we found that TSC DARs are enriched for the RLTR13 family of ERVs, previously identified by profiling of active histone marks in TSCs and containing binding sites for key TSC regulators Cdx2, Eomes and Elf5^9^. Strikingly, however, d2 DARs seem to lack annotated repeat regions. Rather d2 DARs are highly enriched for distinct transcription factor binding motifs such as for Tfap2c, and Ets and GATA sites potentially bound by Elf5 and Gata2 respectively, known to be involved in trophoblast differentiation^31, 43^.

When we compared Blimp1 ChIP-seq datasets from trophoblasts, PGCLCs and SI to genes derepressed in *Prdm1* mutant trophoblasts we found both shared and partially overlapping, as well as cell type-specific targets. For example, *Irak2* and *Mmp2* appear to be common targets in all three cell types, while *Vsig10* appears to be unique to trophoblasts. As for transfected P19 embryonal carcinoma cells^33^, here we observe occupancy by Blimp1 and Tfap2c at common target sites in trophoblasts, though it is not clear whether they bind independently or cooperatively in this case. Tfap2c functions as a transcriptional repressor governing trophoblast differentiation within the SpT layer^29^, but a cooperative Blimp1 and Tfap2c functional relationship has not previously been reported during placenta development. Interestingly, coexpression was recently confirmed in trophoblasts at the single-cell level^20^. Future studies will explore whether Blimp1 and Tfap2c function as transcriptional partners to cooperatively regulate target gene expression in discrete spongiotrophoblast cell lineages.

Identification of direct Blimp1 targets *in vivo* using ChIP approaches is confounded by the diversity of Blimp1+ cell populations^20^. Gene expression changes may also be masked by organ-wide ensemble averaging. The present trophoblast-specific *in vitro* microarray combined with ChIP-seq analysis, however, demonstrates a significant overlap with genes upregulated in Blimp1 mutant placenta at E9.5 (Fig. 7A). Many of these are known to play important functional roles in cell migration and invasion. For example Supervillin (Svil) controls podosome function and turnover^44^. Human invasive extra-villous trophoblasts (EVTs), analogous to mouse SpA-TGCs^45^ form atypical podosomes that regulate extracellular matrix degradation and cell migration^46^. Disabled 2 interacting protein (Dab2ip) inhibits cell invasion in a variety of contexts^47, 48^, and upregulation in human trophoblasts *in vitro* is associated with reduced invasive and migratory abilities^49^. Blimp1 repression of *Dab2ip* may be required to promote trophoblast invasion.

A critical feature of placenta development is specification and migration of the invasive trophoblasts that function in remodelling maternal spiral arteries to increase blood flow to the developing foetus. The genes expressed by this specialized trophoblast subset mediate both vascular mimicry and evasion of the maternal immune system^3, 20, 50^. Many of the 125 Blimp1 targets identified here, however, are either poorly characterized or unstudied in the context of placenta. For example, *Vsig10* is a robust candidate target gene but its functional activities have not been previously reported. However, it is striking that the Blimp1 target genes identified here are significantly enriched for genes with vascular and immunological, as well as cellular signalling functions. For example, it seems likely that Blimp1 controls intracellular signalling via repression of Ras guanyl-nucleotide exchange factors such as *Arhgef18*, as well as the signalling-related kinase *Irak2*, which promotes inflammatory response through NF-kappaB pathway activation^51^. *Arhgef18* has been implicated in cell invasion, which is highly impaired in *Prdm1* mutants^52, 53^. Additionally, Blimp1 regulates expression of matrix metalloproteinase *Mmp2*, implicated in trophoblast invasion and preeclampsia, though its precise function in the placenta requires clarification^54–56^. Multiple other genes, such as candidate tumour suppressor *Vwa5a* ^36^, and the regulator of trophoblast invasion *Cd44* ^37^ may also be key to the *Prdm1* mutant phenotype. Amongst the other identified targets of Blimp1 is the RING domain containing isoform of *Lnx1*, which is reported to ubiquitinate specific isoforms of NUMB, leading to its degradation^35^. NUMB is a multifunctional protein implicated in human trophoblast cell migration and apoptosis^57^. *Numb* mouse mutants die mid-gestation probably due to placental insufficiency, though a detailed characterization of the precise placental defects have not been performed^58^. It is possible that upregulation of *Lnx1* contributes to the *Prdm1* null placental phenotype through degradation of Numb. The extent to which the Blimp1 targets identified here contribute to the *Prdm1* null mutant phenotype will be explored in future studies.

Diverse trophoblast sub-populations that perform essential functions during placental development have been extensively characterized on the basis of location, morphology, and gene expression profiles. Several key transcription factors governing specification of specialized trophoblast sub-types have been identified. For example, Hand1 is required for the formation of trophoblast giant cells at the periphery of the placenta^59^, while Gcm1 is essential for development of the syncytiotrophoblasts that constitute the labyrinth^60^. However the transcriptional regulatory hierarchies guiding cell fate choices remain poorly understood. Here we have characterized Blimp1-dependent target genes and differentially accessible chromatin regions during trophoblast differentiation. Collectively our experiments demonstrate that TSC differentiation protocols in combination with high throughput genomic techniques represents a powerful entry point to identify components of gene regulatory networks that govern later aspects of placenta morphogenesis.

## Methods

### TSC maintenance and differentiation

*Prdm1* ^+/+^ and *Prdm1* ^*BEH/BEH*^ TSC lines isolated previously^20^ were grown under defined conditions^61^. To promote differentiation trypsinized cultures were plated in the absence of FGF4, TGFβ1 and heparin sulfate.

### Microarray data analysis

Published Illumina Mouse WG-6 v2 Expression BeadChip microarray data corresponding to four independently derived *Prdm1* ^+/+^ and *Prdm1* ^*BEH/BEH*^ null mutant TSC lines (NCBI GEO accession number GSE74409) were analysed as previously described^20^. Further analysis using K-means clustering was performed using Cluster 3.0^62^. Gene set enrichment analysis (GSEA)^63, 64^ was performed using all genes represented on the microarray pre-ranked on Illumina DiffScore derived from the differential expression analysis and compared with the nearest gene transcription start site ± 100 kb from each Blimp1 ChIP-seq peak. For maximum stringency where multiple gene sets are tested we report both nominal *P* value and family-wise error rate (FWER) *P* value.

For gene expression comparison with TSCs published Illumina Mouse WG-6 v2 Expression BeadChips microarray data for ESCs was downloaded from NCBI (GEO accession number GSE46308). After background subtraction using GenomeStudio v2009 (Illumina) raw data were quantile normalized and differentially expressed genes identified using ArrayAnalysis^65, 66^. Comparison with gene sets defined by ATAC-seq analysis was performed on quantile normalized data using GSEA with standard parameters.

### ATAC-seq analysis

ATAC-seq libraries from two independent wild type cell lines were generated for TSCs and day 2 of differentiation (d2) as described^21, 67^ using 75,000 cells per replicate. Multiplexed 75 bp paired-end sequence reads generated on a single lane of an Illumina HiSeq. 4000 were mapped to the mm10 mouse genome build using Stampy with default parameters^68^. Mapped data were visualized using Integrated Genome Viewer v2.3.88^69^. Regions of open chromatin were identified by MACS2 using default parameters^70^. Regions detectably accessible in both wild type cell lines were identified by overlapping peak coordinates and used for subsequent analyses (Supplementary Fig. S1). To identify high confidence differentially accessible chromatin regions (DARs) between TSCs and d2 trophoblasts a MACS2 q-value cutoff of 1 × 10^−5^ was applied. DARs consistently detected in both cell lines were then identified and used for subsequent analyses (Supplementary Fig. S1). ATAC-seq data were deposited in NCBI GEO under accession number GSE94694.

For comparisons with TSC ATAC-seq data 8-cell embryo and ESC ATAC-seq data mm9 peak regions were downloaded from NCBI GEO accession number GSE66581 and converted to mm10 coordinates using liftOver^71^.

### *De novo* motif analysis

*De novo* motif analysis was performed using MEME-ChIP with default parameters^72^. Matches to known motifs were identified using TOMTOM^73^. Frequency of *de novo* identified Blimp1 binding motif within peaks was performed with FIMO^74^ using a *P* value cutoff of 1 × 10^−4^.

### Analysis of repeat elements

Repeat element annotations for the GRCm38/mm10 genome build were downloaded from the UCSC Genome Browser database^75, 76^ and overlap with ATAC-seq peaks was performed using custom Perl scripts. For comparison repeat annotations were also overlapped with a set of 10,000 randomly generated genomic regions of equivalent size to the ATAC-seq peaks. Statistical differences were determined using Chi-square with Yates’ correction.

### ChIP-seq analysis

D2 cells from two independent *Prdm1* ^+/+^ and *Prdm1* ^*BEH/BEH*^ TSC lines each were fixed for 15 minutes at room temperature using 1% formaldehyde in culture medium. Samples were then processed for ChIP-seq as previously described^77^ using 4 × 10^7^ cells per replicate and 14 µg of mouse IgG1 anti-Blimp1 ascites fluid (Novus, clone 3H2-E8, Lot # 102612)^78^. 51 bp paired-end reads generated by multiplexing ChIP and associated input samples on 2 lanes of an Illumina HiSeq. 2500 were mapped to the mm10 mouse genome build with Bowtie2^79, 80^ in Galaxy^81^ using default parameters, except –k 2. Peaks were called for each ChIP sample relative to its input using MACS2^70^ with default parameters. Non-specific peaks detectable in *Prdm1* ^*BEH/BEH*^ mutant cells were subtracted from wild type datasets (Supplementary Fig. S8). Substantial overlap was observed between wild type replicates, however as one replicate contained stronger signal it was selected for all subsequent analyses. Peaks present in both replicates are indicated in Supplementary Table S7 and Supplementary Figure S8. Heatmap comparisons of Blimp1 ChIP-seq coordinates with ATAC-seq data was performed using seqMINER v1.3.3^82^. ChIP-seq data was deposited in NCBI GEO under accession number GSE74408.

Tfap2c d1 trophoblast ChIP-seq data was downloaded from the European Nucleotide Archive (accession PRJNA298763) and analysed as above using a MACS2 q-value cut-off of 1 × 10^−15^. Blimp1 ChIP-seq data from E18.5 small intestine and PGCLCs mm9 peak coordinates were downloaded from the published papers^18, 34^ and converted to mm10 using liftOver^71^. Esrrb ChIP-seq coordinates (mm10) were downloaded from the published paper^32^.

H3K4me1, H3K4me3, H3K9me4, H3K27me3 and H3K27ac TSC ChIP-seq data was downloaded from NCBI GEO accession GSE 42207 and mapped to the mm10 genome build using Bowtie2 and compared with ATAC-seq coordinated using seqMINER as above.

### Comparison of ATAC-seq and ChIP-seq coordinates

To determine whether overlaps of sets of ChIP-seq and ATAC-seq peaks were statistically significant we applied a multi-step process. First we characterized the pairwise overlap of all peaks within datasets being compared, relative to 1,000 iterations of random regions of equivalent size using GAT^83^. Plots indicating significance per peak are given in Supplementary Figure S2. Next, for significantly overlapping peaks we asked whether the number of peaks with ≥ the median percentage overlap per dataset was greater than expected by chance based on 1,000 iterations of random regions of equivalent size using Chi-square test in Yates’ correction. *P* values per dataset comparison and percentage overlap per significantly overlapping peak are given in Supplementary Figure S2.

### Functional annotation analysis

Functional annotation analysis of ATAC-seq and ChIP-seq peak datasets was performed using GREAT version 3.0.0^84^, linking peaks to the nearest transcription start site (TSS) ± 100 kb. Functional annotation of gene lists derived from microarray analysis was performed using Enrichr^85, 86^. Non-redundant functional terms were selected based on reported significance score and relevance to the biological system.

### qRT-PCR

RNA was extracted using RNeasy mini kits (QIAGEN) according to manufacturers instructions. Quantitative PCR (qPCR) was performed as previously described^87^ using *Actb* to normalize gene expression. Primer sequences used are shown in Supplementary Table S8. Changes in gene expression were determined using the 2^−∆∆CT^ method with expression represented as mean ±S.E.M. Statistical significance was determined using two-tailed homoscedastic t-test.

### Data availability

ChIP-seq and ATAC-seq data have been deposited in NCBI GEO (accession numbers GSE74408 and GSE94694 respectively). Details of published datasets used in this study are provided in Methods.

## Electronic supplementary material

Supplementary Information

Table S1

Table S2

Table S3

Table S4

Table S5

Table S6

Table S7

Table S8
