# Supplementary material for: Mapping the chromatin landscape and Blimp1 transcriptional targets that regulate trophoblast differentiation
Source: Sci Rep. 2017 Jul 28;7:6793. doi: 10.1038/s41598-017-06859-9 (PMC5533796; doi:10.1038/s41598-017-06859-9)
Supplement: Supplementary file 1 — Supplementary Information [file 41598_2017_6859_MOESM1_ESM.pdf]

Supplementary Information

Mapping the chromatin landscape and Blimp1 transcriptional targets that regulate trophoblast differentiation

Andrew C. Nelson[1,2], Arne W. Mould[1], Elizabeth K. Bikoff[1], and Elizabeth J. Robertson[1]

1. Sir William Dunn School of Pathology, University of Oxford, South Parks Road, Oxford, OX1 3RE, UK.
2. School of Life Sciences, Gibbet Hill Campus, University of Warwick, Coventry, CV4 7AL, UK.

Supplementary Figures and Figure Legends

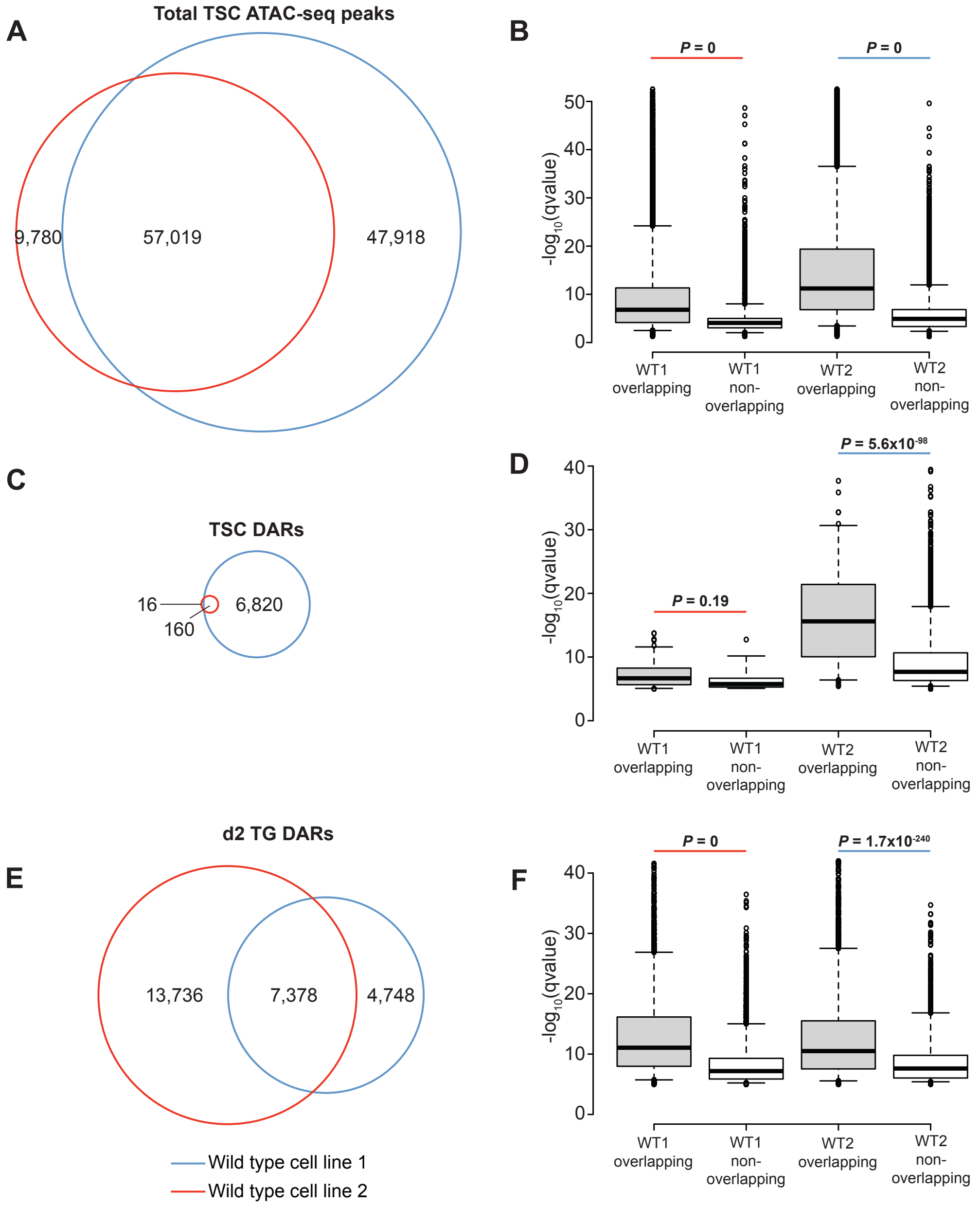

Figure S1. Overlap of TSC ATAC-seq peaks and TSC and d2 DARs identified in each cell line.

(A) Venn diagram indicating the degree of overlap between TSC ATAC-seq peaks from two independent TSC lines. Note that overlapping peaks were used for subsequent analyses in the main paper.

(B) ATAC-seq peaks identified in both TSC lines show greater significance values, suggesting that a subset of peaks are not detected in both cell lines due to being near the applied significance threshold. Heteroscedastic t-test *P* values are indicated.

(C) Venn diagram indicating the degree of overlap between TSC DARs peaks identified in two independent TSC lines. Note that overlapping DARs were used for subsequent analyses in the main paper. Validation of the trends in

both the overlapping and non-overlapping TSC DARs are shown in Supplementary Figure S5.

(D) TSC DARs identified in both cell lines show greater significance values, suggesting that a subset of DARs are not detected in both cell lines due to being near the applied significance threshold. Heteroscedastic t-test *P* values are indicated.

(E) Venn diagram indicating the degree of overlap between d2 DARs peaks identified in two independent TSC lines. Note that overlapping DARs were used for subsequent analyses in the main paper.

(F) D2 DARs identified in both cell lines show greater significance values, suggesting that a subset of DARs are not detected in both cell lines due to being near the applied significance threshold. Heteroscedastic t-test *P* values are indicated.

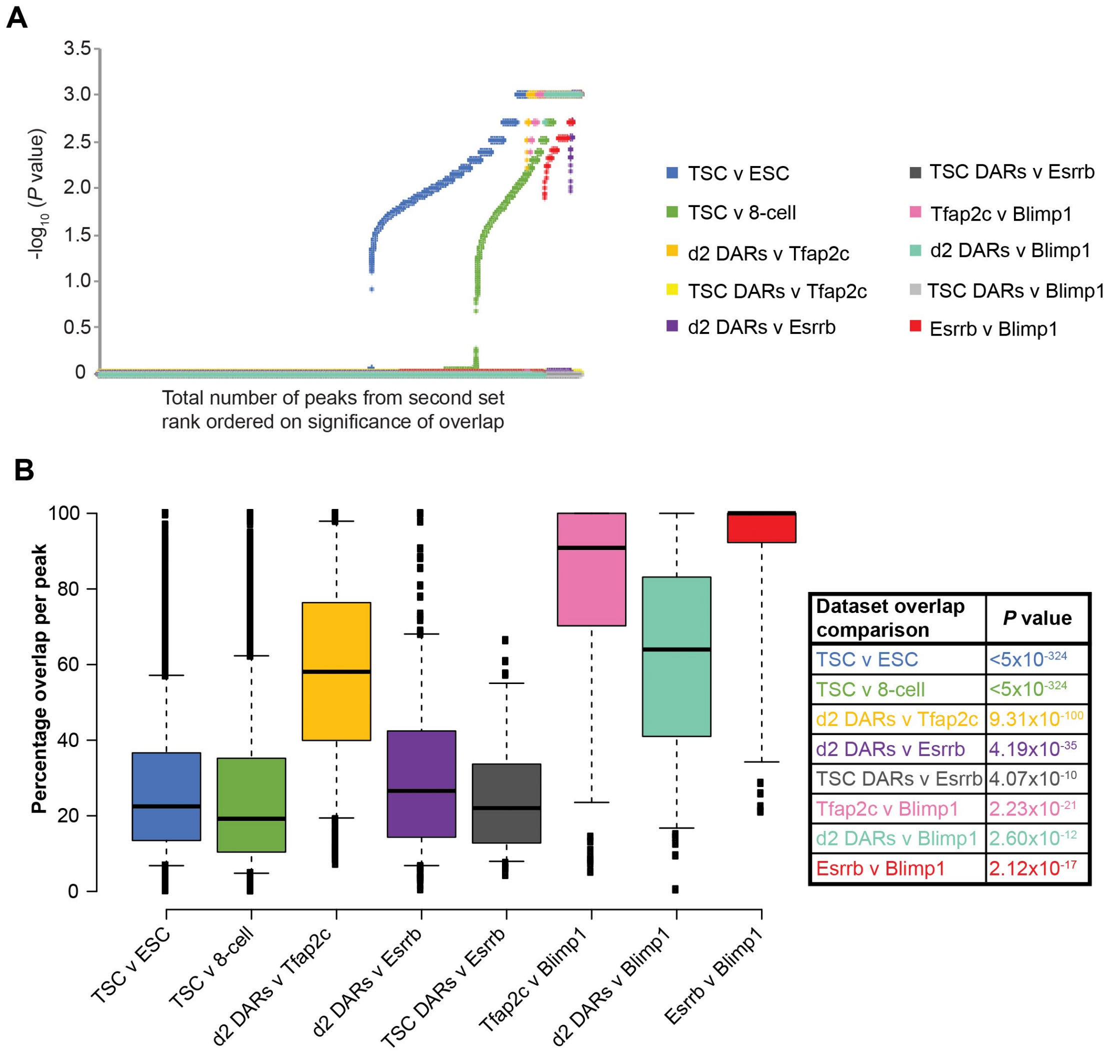

Dataset overlap comparison	*P* value
TSC v ESC	$<5\times10^{-324}$
TSC v 8-cell	$<5\times10^{-324}$
d2 DARs v Tfap2c	9.31×10^{-100}
d2 DARs v Esrrb	4.19×10^{-35}
TSC DARs v Esrrb	4.07×10^{-10}
Tfap2c v Blimp1	2.23×10^{-21}
d2 DARs v Blimp1	2.60×10^{-12}
Esrrb v Blimp1	2.12×10^{-17}

Figure S2. Assessment of significance of overlaps between ATAC-seq and ChIP-seq datasets.

(A) Scatter plot indicating the per peak significance of overlap ordered from least to most significant for the second dataset indicated at each key label. Significance calculation is based on 1,000 iterations of overlapping random genomic regions of equivalent size – see Methods. Relates to Venn diagrams in Figures 1A, 3G, 4C, 5B, 8H and 8I. Note that most peaks do not overlap but are represented in the plot.

(B) Left: boxplots of percentage of overlap per peak for significantly overlapping peaks, relative to the second dataset indicated at each axis label. Box and whisker intervals are 5th, 25th, 50th, 75th and 95th percentiles. Right: Table of *P* values from Chi-squared test with Yates' correction indicating the likelihood that the number of significant peaks with greater than the median overlap occurred at a greater frequency than expected by chance based on 1,000 iterations of overlapping random genomic regions of equivalent size.

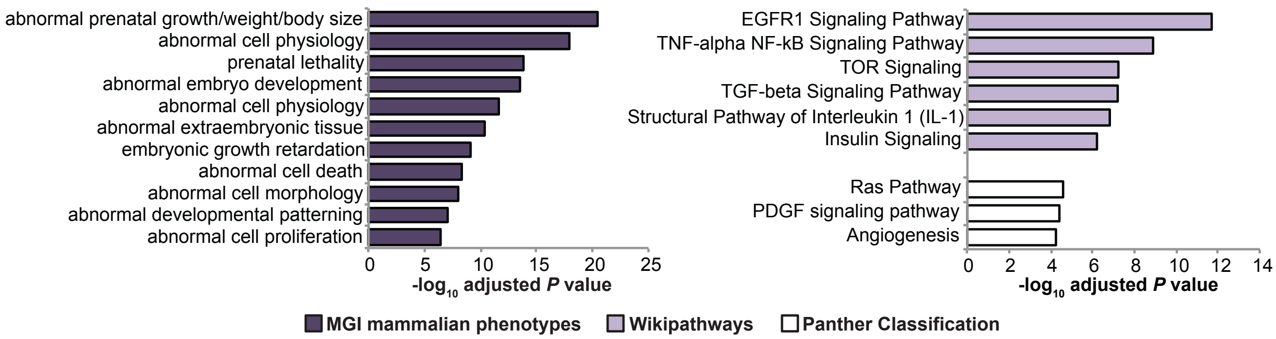

Figure S3. Functional annotation analysis of genes from each significant gene set identified by GSEA leading edge analysis in Figure 1B (core genes with TSC ATAC-seq peaks and significantly higher expression in TSCs).

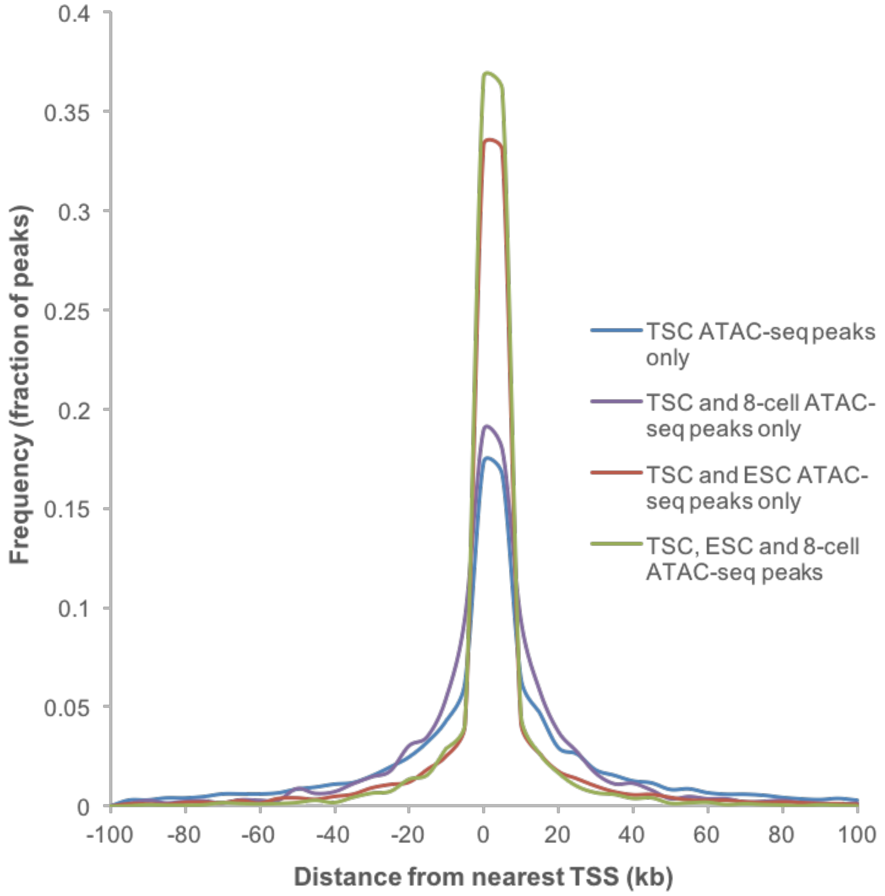

Figure S4. Distribution of TSC ATAC-seq peak subsets overlapping 8-cell and ESC data as defined in Figure 1A. Note that a higher proportion of TSC peaks overlapping 8-cell peaks are at greater distances from the nearest TSS, consistent with being enhancers (Fig. 1F).

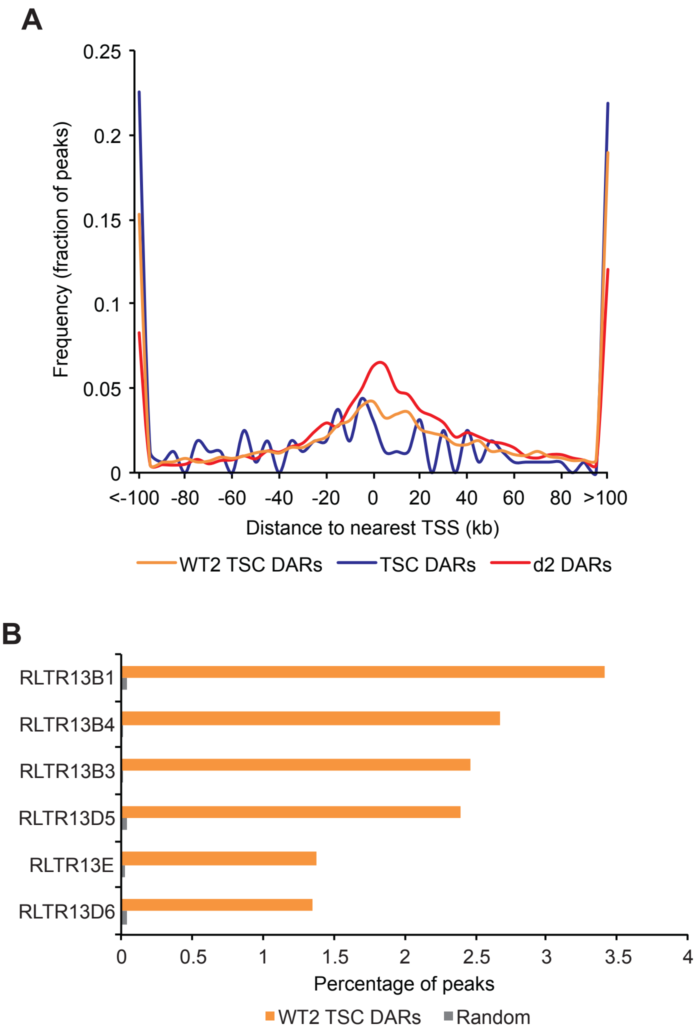

Figure S5. Validation of trends in TSC DARs from cell line WT2 compared to TSC DARs identified in both cell lines (See Fig. S1C).

(A) TSC DARs from cell line WT2 are significantly further from TSSs than d2 DARs – $P = 1.8\text{x}10^{-105}$, two-tailed homoscedastic t-test (see Fig. 2D).

(B) TSC DARs from cell line WT2 are enriched for RLTR13 family endogenous retroviral sequences (see Fig. 3C).

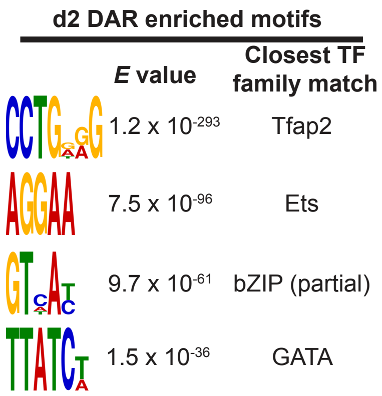

Figure S6. Sequence motifs identified in d2 DARs using MEME-ChIP.
E value confidence scores and the closest transcription factor family match identified by TOMTOM are shown.

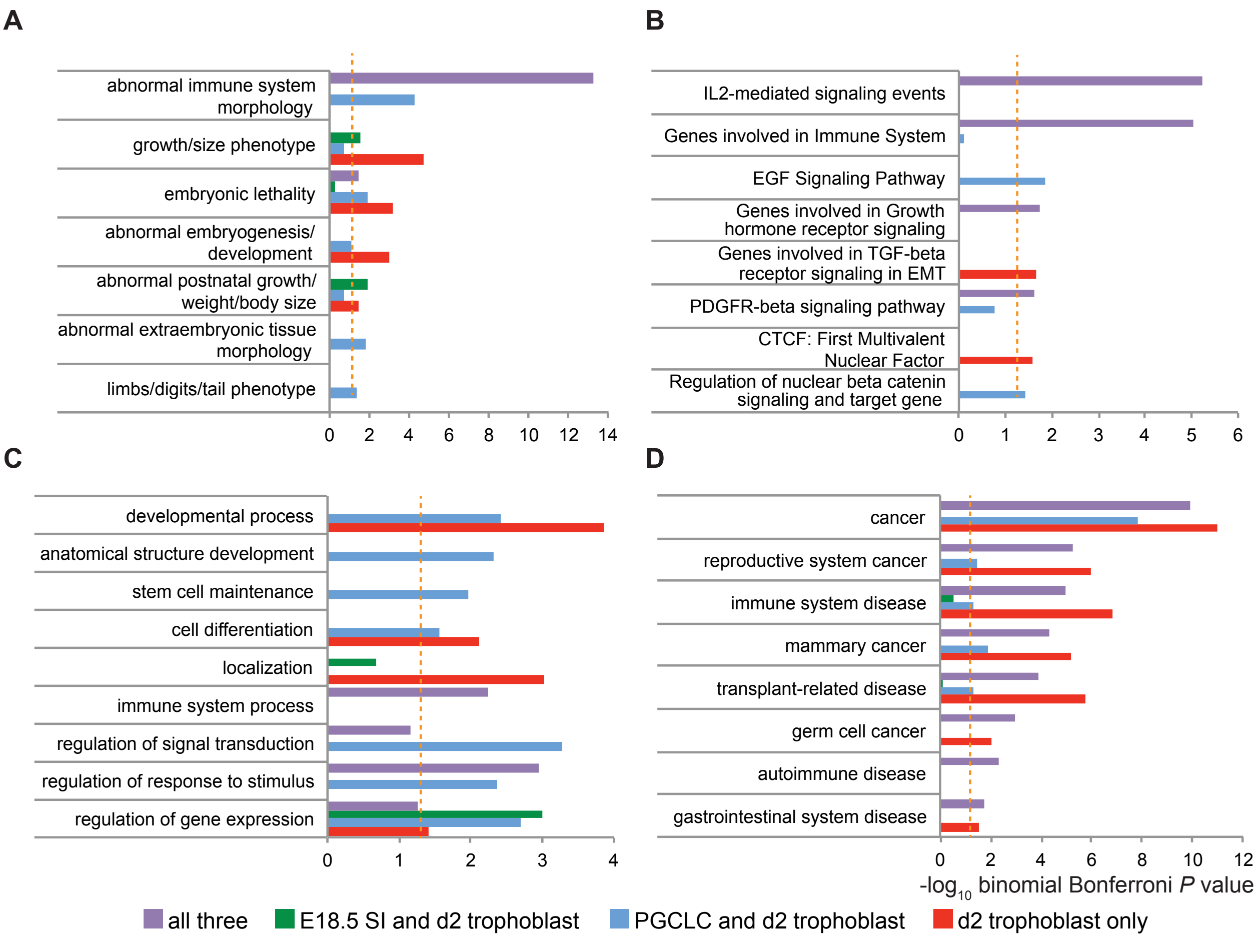

Figure S7. Common and distinct functions of Blimp1 in different cell types.

(A) Bar graph of selected MGI Phenotype terms associated with peaks indicated in Figure 5B identified using GREAT.

(B) Selected MSigDB Pathway terms.

(C) Selected Gene Ontology Biological Process terms.

(D) Selected Disease Ontology terms. Dashed orange line indicates binomial Bonferroni *P* value of 0.05.

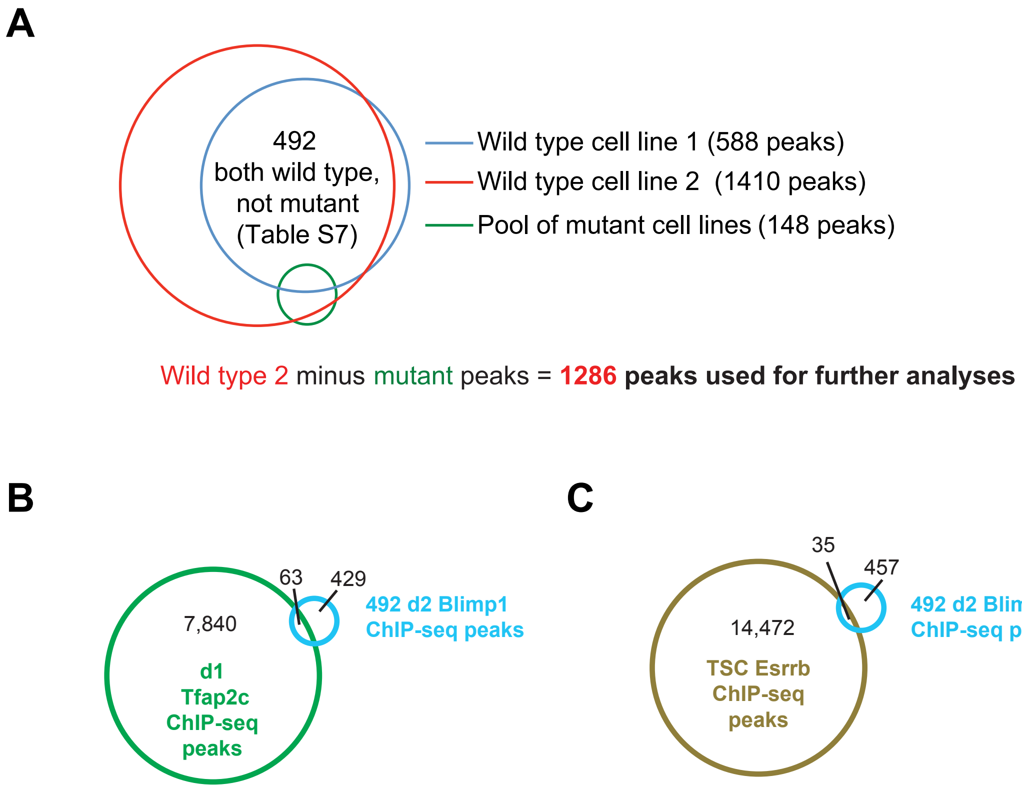

Figure S8. Overlap of Blimp1 ChIP-seq replicates and control.

(A) Venn diagram indicating numbers of ChIP-seq peaks identified using mouse anti-Blimp1 antibody in two independent differentiated wild type TSC lines and *Prdm1* mutant cells.

(B) Overlap of the 492 d2 Blimp1 ChIP-seq peaks identified in both replicates with and d1 Tfap2c ChIP-seq peaks. See also Figure 5B. Note that 12.8% of the core 492 Blimp1 peaks overlap with d1 Tfap2c peaks, compared with 10.7% of the larger 1286 wild type 2 peak set (P value = 3.54×10^{-210}, Chi-squared tests with Yates' correction).

(C) Overlap of the 492 d2 Blimp1 ChIP-seq peaks identified in both replicates with and TSC Esrrb ChIP-seq peaks. See also Figure 8I. Note that 7.1% of the core 492 Blimp1 peaks overlap with TSC Esrrb peaks, compared with 7.9% of the larger 1286 wild type 2 peak set (P value = 0.67, Chi-squared tests with Yates' correction).

Supplementary Tables

Table S1. TSC ATAC-seq peaks with nearest TSSs ±100kb and overlapping 8 cell and ESC ATAC-seq peaks indicated.

Table S2. ESC vs. TSC microarray data.

Table S3. TSC ATAC-seq differentially accessible regions (DARs) with nearest TSSs ±100kb and overlapping Esrrb and Tfap2c ChIP-seq peaks indicated.

Table S4. D2 ATAC-seq differentially accessible regions (DARs) with nearest TSSs ±100kb and overlapping Esrrb and Tfap2c ChIP-seq peaks indicated.

Table S5. D2 Blimp1 ChIP-seq peaks with nearest TSSs ±100kb and overlapping PGCLC and E18.5 small intestine Blimp1 ChIP-seq peaks, Tfap2c and Esrrb ChIP-seq peaks, and d2 ATAC-seq DARs indicated.

Table S6. Blimp1 ChIP-seq peaks and microarray expression profiles for the 125 direct target genes

Table S7. D2 Blimp1 ChIP-seq peaks identified in both wild type TSC lines with IDs as in Table S5.

Table S8. Primers used for qRT-PCR analysis
